# Choice of reference-guided sequence assembler and SNP caller for analysis of *Listeria monocytogenes* short-read sequence data greatly influences rates of error

**DOI:** 10.1186/s13104-015-1689-4

**Published:** 2015-12-08

**Authors:** Arthur W. Pightling, Nicholas Petronella, Franco Pagotto

**Affiliations:** Office of Analytics and Outreach, Center for Food Safety and Applied Nutrition, U.S. Food and Drug Administration, 5100 Paint Branch Parkway, College Park, MD 20740 USA; Biostatistics and Modelling Division, Bureau of Food Surveillance and Science Integration, Food Directorate, Health Products and Food Branch, Health Canada, 251 Sir Frederick Banting Driveway, Ottawa, ON K1A 0K9 Canada; Listeriosis Reference Service for Canada, Microbiology Research Division, Bureau of Microbial Hazards, Food Directorate, Health Products and Food Branch, Health Canada, 251 Sir Frederick Banting Driveway, Ottawa, ON K1A 0K9 Canada

**Keywords:** SNP detection, Short-read sequence assembly, Reference-guided sequence assembler, Reference sequence, Sequencing coverage, *Listeria monocytogenes*, 08-5578, HB5622, EGD-e, SNP caller

## Abstract

**Background:**

The influences that different programs and conditions have on error rates of single-nucleotide polymorphism (SNP) analyses are poorly understood. Using Illumina short-read sequence data generated from *Listeria monocytogenes* strain HPB5622, we assessed the performance of four SNP callers (BCFtools, FreeBayes, UnifiedGenotyper, VarScan) under a variety of conditions, including: (1) a range of sequencing coverages; (2) use of four popular reference-guided assemblers (Burrows-Wheeler Aligner, Novoalign, MOSAIK, SMALT); (3) with and without read quality trimming and filtering; and (4) use of different reference sequences.

**Results:**

At 8-fold coverage the proportions of true positive calls ranged from 0.22 to 25.00 % when reads were aligned to a nearly identical reference (0.000096 % distant). Calls made when reads were aligned to a non-identical reference (0.85 % distant) were from 92.54 to 98.88 % accurate. At 79-fold coverage accuracies ranged from 3.95 to 20.00 % with the nearly identical reference and 93.80–98.75 % with the non-identical reference. Read preprocessing significantly changed the numbers of false positive calls made, from a 65.24 % decrease to a 54.55 % increase.

**Conclusions:**

The combinations of reference-guided sequence assemblers and SNP callers greatly influenced not only the numbers of true and false positive sites but also the proportions of true positive calls relative to the total numbers of calls made. Furthermore, the efficacy of different assembler and caller combinations changed dramatically with the different conditions tested. Researchers should consider whether identifying the greatest numbers of true positive sites, reducing the numbers of false positive calls, or achieving the highest accuracies are desired.

**Electronic supplementary material:**

The online version of this article (doi:10.1186/s13104-015-1689-4) contains supplementary material, which is available to authorized users.

## Background

Next-generation sequencing of bacterial genomes is an increasingly valuable tool in a number of fields, including epidemiology [1–3], population genetics [4, 5], and experimental evolution [6]. Reduced sequencing costs [7] and a wide availability of open-source software have made assembling and analyzing whole-genome sequence data more accessible than ever [8]. Comparative analyses in which nucleotide differences (single nucleotide polymorphisms or, simply, SNPs) between a subject and reference are identified can be particularly useful for distinguishing bacterial lineages [9] and may provide markers for phenotypes such as antibiotic resistance [10]. SNPs are usually identified by first using reference-guided sequence assembly software to align large numbers of short sequence reads to a fully sequenced (closed) reference chromosome or plasmid sequence [11]. Then, additional programs (SNP callers) are used to analyze assemblies and identify differences between the reference and draft genome sequences by using a combination of sequence coverage and read quality information [12, 13]. Importantly, different SNP callers use different algorithms and assumptions that are likely to influence the accurate identification of SNPs.

Inaccuracies in sequence assemblies can arise due to a combination of the short length of sequence reads (for example, ~200–250 bp for Illumina sequencing) and inherent errors associated with sequencing technologies [14] (possibly influenced by the quality of DNA extractions and library preparations [15]). In addition, low sequencing coverage [16] and the use of genetically distant reference sequences [17] provide additional computational challenges for both reference-guided sequence assemblers and SNP callers [18], especially around regions of repeated DNA sequence [19]. These issues may result in diminished detection of true SNP differences (true positive calls) and increased numbers of misidentified SNPs (false positive calls). In order to mitigate errors, read quality trimming and filtering prior to assembly may be performed [20]. However, while some researchers have reported benefits from such preprocessing [18], others have demonstrated that trimming and filtering did not improve the accuracy of SNP calls [21]. Thus, several factors may influence the accurate identification of true nucleotide differences: (1) sequencing coverage, (2) read preprocessing, (3) availability of an appropriate reference sequence, (4) selection of short-read sequence assembler, and (5) one’s choice of SNP calling software.

We assessed the efficacy of SNP calling programs by generating next-generation sequence datasets of varying quality, assembling and analyzing the resulting reads under a variety of conditions, and by counting the numbers of true and false positive calls that were made under different conditions. We isolated genomic DNA from the Listeriosis Reference Service for Canada’s (LRS) *Listeria monocytogenes* strain HPB5622 isolate and generated eight sets of Illumina short-read sequence data with a MiSeq benchtop sequencer (Illumina, San Diego). *L. monocytogenes* is a Gram-positive pathogenic bacterium [22] that experiences few chromosomal rearrangements [23, 24]. We then assembled and analyzed the resulting reads under a variety of conditions: (1) a range of sequencing coverages; (2) the use of four popular reference-guided assemblers (Burrows-Wheeler Aligner [25], Novoalign, MOSAIK, and SMALT) that use different algorithms for assembly (Burrows-Wheeler transform [26], global Needleman-Wunsch [27], banded Smith-Waterman, and a combination of short-word hashing and Smith-Waterman [28, 29], respectively); (3) with and without read quality trimming and filtering prior to assembly; (4) the use of reference sequences of different genetic distances; and (5) the use of different SNP callers (BCFtools [30], FreeBayes [31], UnifiedGenotyper (https://www.broadinstitute.org/gatk/gatkdocs/org_broadinstitute_gatk_tools_walkers_genotyper_UnifiedGenotyper.php), and VarScan [32, 33]). We assembled each dataset using both *L. monocytogenes* strain 08-5578 [2, 3] and EGD-e [34] chromosome sequences obtained from the National Center for Biotechnology archive as references. Strains 08-5578 and EGD-e are approximately 0.000096 and 0.82 % distant from the HPB5622 chromosome sequence at the nucleotide level, respectively.

## Results and discussion

We assessed the ability of four commonly used single-nucleotide polymorphism (SNP) callers (BCFtools, FreeBayes, UnifiedGenotyper, and VarScan) to identify SNPs from alignments of eight sets of *Listeria monocytogenes* strain HPB5622 genomic DNA sequence data of varying quality generated on an Illumina MiSeq benchtop sequencer. All sequencing runs were performed on genomic DNA obtained from a single extraction. Performance was measured by counting the numbers of known nucleotide differences between the subjects and references (true positive sites) correctly identified and the numbers of incorrect calls made (false positive sites) with each SNP caller under a variety of conditions. We assembled each of the short-read sequence datasets with the Burrows-Wheeler Aligner (BWA) using reference chromosome sequences (08-5578 and EGD-e) obtained from the National Center for Biotechnology Information (NCBI) sequence database (NC_013766.1 [2] and NC_003210.1 [34], respectively). The Listeriosis Reference Service for Canada’s (LRS) strain HPB5622 isolate differs from the strain 08-5578 sequence at three nucleotide positions (1,3629,720; 2,870,261; and 2,870,308). In addition, the HPB5622 chromosome sequence differs from the strain EGD-e sequence at 24,890 nucleotide positions. Thus, we were able to measure the numbers of true and false positive sites identified by SNP callers when a nearly identical (~0.000096 % distant at the nucleotide level) and a non-identical (~0.85 % distant) chromosome sequence was used for short-read sequence assembly.

**Fig. 1:**
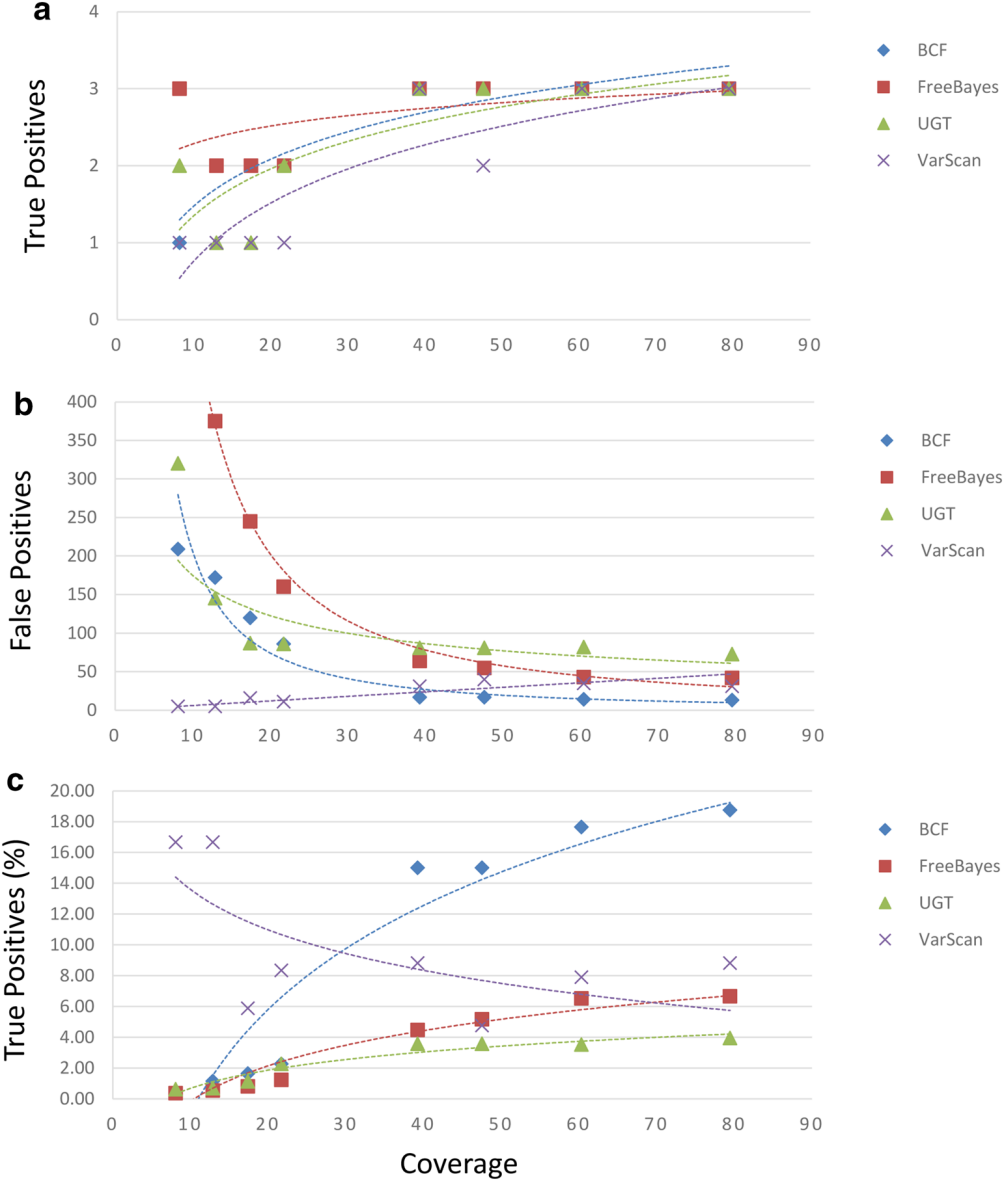
Comparison of SNP calls calculated from alignments of Illumina reads to a nearly identical reference. Genomic DNA from the Listeriosis Reference Service for Canada’s (LRS) *Listeria monocytogenes* strain HPB5622 culture was indexed and sequenced eight times. The resulting reads were aligned with the Burrows-Wheeler Aligner using an *L. monocytogenes* strain 08-5578 chromosome sequence obtained from the National Center for Biotechnology Information (NCBI) archive as a reference. The 08-5578 chromosome sequence differs from the HPB5622 chromosome at three nucleotide positions. Four SNP-callers (BCFtools [BCF], FreeBayes, UnifiedGenotyper [UGT], and VarScan) were used to identify nucleotide differences. The numbers of true positive (**a**), false positive (**b**), and the proportions of calls made that correctly identified true positive sites (**c**) relative to the calculated coverages of assemblies are shown

We began by counting the numbers of true and false positive SNP calls made by SNP callers when the Burrows-Wheeler Aligner was used to assemble sequence data from all eight sets of reads. When sequence data was aligned to the strain 08-5578 chromosome sequence we observed that BCFtools, UnifiedGenotyper, and VarScan detected between 1 and 3 true positive sites in 8 assemblies and averaging between 1.88 and 2.38 sites, generally correlating with sequence coverage (Fig. 1, Additional file 1). FreeBayes performed best under these conditions with between 2 and 3 sites correctly identified with an average of 2.63 sites (Fig. 1a). When sequence assemblies of at least 50-fold coverage are considered each SNP caller identified all 3 true positive SNPs. We observed also that between 5 and 818 false positive sites were reported, averaging between 21.75 (VarScan) and 225.25 (FreeBayes; Fig. 1b). When runs of at least 50-fold coverage are considered the average numbers of false positive sites range from 13.50 (BCFtools) to 77.50 (UnifiedGenotyper; Fig. 1c). When we considered the accuracy of the SNP callers by calculating the proportions of true positive sites called to the total numbers of sites called, we calculated that SNP callers were between 2.42 (UnifiedGenotyper) and 9.73 % (VarScan) accurate, with runs of at least 50-fold coverage yielding 3.74 (UnifiedGenotyper) and 18.20 % (BCFtools) correct calls (Fig. 1c). Interestingly, we observed an inverse relationship between identification of false positive sites and sequence coverage when VarScan was used. At approximately eightfold coverage we observed 5 such sites and at approximately 79-fold coverage we observed 31 sites. Thus, we observed that although VarScan is the most accurate SNP caller when coverage is low, due to reductions in false positive calls, BCFtools is the most accurate SNP caller when coverage is high.

**Fig. 2:**
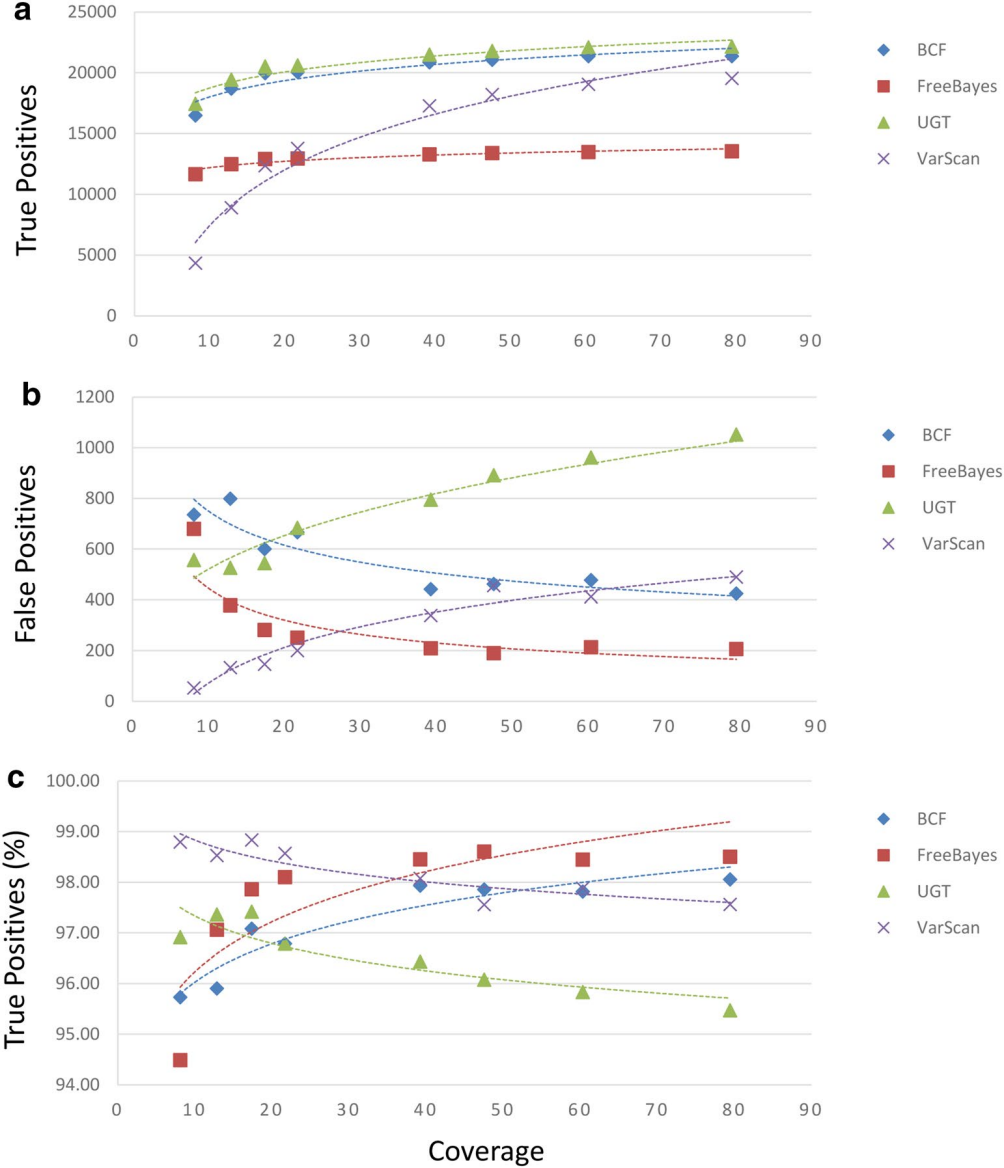
Comparison of SNP calls calculated from alignments of Illumina reads to a non-identical reference. Genomic DNA from the Listeriosis Reference Service for Canada’s (LRS) *Listeria monocytogenes* strain HPB5622 culture was indexed and sequenced eight times. The resulting reads were aligned with the Burrows-Wheeler Aligner using an *L. monocytogenes* strain EGD-e chromosome sequence obtained from the National Center for Biotechnology Information (NCBI) archive as a reference. The EGD-e chromosome sequence differs from the HPB5622 sequence at 24,890 nucleotide positions. Four SNP-callers (BCFtools [BCF], FreeBayes, UnifiedGenotyper [UGT], and VarScan) were used to identify nucleotide differences. The numbers of true positive (**a**), false positive (**b**), and the proportions of calls made that correctly identified true positive sites (**c**) relative to the calculated coverages of assemblies are shown

When a strain EGD-e chromosome sequence was used to align the short-read sequence data, we observed true positive averages ranging from 12,960.38 to 20,692.13 for all datasets and from 13,509.00 to 22,124.00 for runs of at least 50-fold coverage (FreeBayes and UnifiedGenotyper, respectively, in each case; Fig. 2a, Additional file 2). The average numbers of false positive sites observed ranged from 278.50 (VarScan) to 751.13 (UnifiedGenotyper) and 209.50 (FreeBayes) to 1006.50 (UnifiedGenotyper) for alignments of 50-fold coverage greater (Fig. 2b). The accuracies of SNP callers was measured between 96.54 (UnifiedGenotyper) and 98.22 % (VarScan). For runs of at least 50-fold coverage between 95.65 (UnifiedGenotyper) and 98.47 % (FreeBayes) accuracies were observed (Fig. 2c). While both the UnifiedGenotyper and VarScan demonstrated an inverse correlation between sequence coverage and false positive sites, VarScan, once again, had the greatest accuracy among low coverage sequencing runs.

**Fig. 3:**
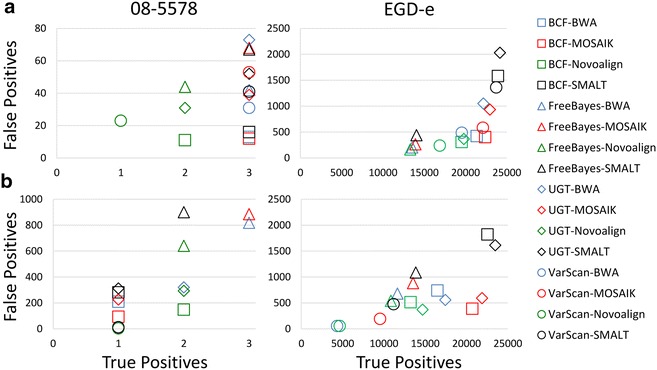
Comparison of 16 combinations of reference-guided sequence assemblers and SNP callers. Genomic DNA from the Listeriosis Reference Service for Canada’s (LRS) *Listeria monocytogenes* strain HPB5622 culture was indexed and sequenced, yielding a high (~79-fold) and a low (~eightfold) coverage datasets. The resulting reads were aligned with the Burrows-Wheeler Aligner (BWA), MOSAIK, Novoalign, and SMALT using both *L. monocytogenes* strain 08-5578 (**a**) and EGD-e (**b**) chromosome sequences obtained from the National Center for Biotechnology Information (NCBI) archive as references. The NCBI strain 08-5578 chromosome sequence differs from HPB5622 at three nucleotide positions, while the EGD-e chromosome sequence differs at 24,890 nucleotide positions. Four SNP-callers (BCFtools [BCF], FreeBayes, UnifiedGenotyper [UGT], and VarScan) were used to identify nucleotide differences

We counted the numbers of true and false positive sites observed using 16 different combinations of reference-guided sequence assemblers and SNP callers (BCFtools, FreeBayes, UnifiedGenotyper, and VarScan with BWA, MOSAIK, Novoalign, and SMALT) with both strain 08-5578 and EGD-e chromosomes as references and with high and low levels of sequence coverage (Fig. 3, Additional file 3). When reads from the high sequence coverage dataset were aligned to the strain 08-5578 chromosome (Fig. 3a) we observed that all the three true positive SNPs were correctly identified by each SNP caller when either BWA, MOSAIK, or SMALT were used, while assemblies generated by Novoalign resulted in only 1 (VarScan) or 2 (BCFtools, FreeBayes, and UnifiedGenotyper) correct calls. In addition, we observed between 11 (BCFtools-Novoalign) and 73 (UnifiedGenotyper-BWA) false positive calls. The percentages of the total numbers of calls made that were correct (i.e., true positive) ranged from 3.95 (UnifiedGenotyper-BWA) to 20.00 % (BCFtools-MOSAIK). More generally, use of BCFtools resulted in substantially fewer false positive calls (average 13.00) regardless of which sequence assembler was used.

When reads from the high coverage dataset were aligned to the strain EGD-e chromosome (Fig. 3a) we observed that between 13,304 and 24,164 true positive sites were identified and from 168 to 2032 incorrect calls were made (FreeBayes-Novoalign and UnifiedGenotyper-SMALT, respectively, in both cases). We also observed accuracies ranging from 92.24 (UnifiedGenotyper-SMALT) to 98.75 % (FreeBayes-Novoalign). Thus, we observed a correlation, albeit a non-linear one, between the numbers of true and false positive calls made, with FreeBayes-Novoalign resulting in both the fewest true and false positive calls and UnifiedGenotyper-SMALT resulting in both the greatest numbers of true and false positive calls. We also observed that although the UnifiedGenotyper-SMALT combination resulted in the identification of nearly all true positive sites, the greater number of false positive calls resulted in low accuracy, while the FreeBayes-Novoalign combination has the highest accuracy, despite the fact that only about half of the total numbers of true nucleotide differences were correctly identified.

We assembled reads from a low coverage dataset by aligning them to the strain 08-5578 chromosome sequence (Fig. 3b, Additional file 3). Only two combinations of reference-guided sequence assemblers and SNP callers (FreeBayes-BWA and FreeBayes-MOSAIK) resulted in the correct identification of all three nucleotide differences. The numbers of false positive sites ranged from 3 (VarScan-Novoalign) to 900 (FreeBayes-SMALT). Indeed, VarScan consistently called the fewest numbers of false positive sites and FreeBayes consistently called the greatest numbers of false positive sites, no matter which assembler was used. The frequencies of true positive calls ranged from 0.31 (FreeBayes-Novoalign) to 25.00 % (VarScan-Novoalign). Again, we observed a pattern in which VarScan-Novoalign only correctly identified one true positive SNP but, due to a paucity of false positive calls, demonstrated the highest degree of accuracy.

We reassembled the low coverage dataset by aligning reads to the strain EGD-e chromosome sequence (Fig. 3b, Additional file 3). Between 4,340 (VarScan-BWA) and 23,504 (UnifiedGenotyper-SMALT) true positive sites were properly identified, while between 53 (VarScan-BWA and VarScan-Novoalign) false positive sites were called. In addition, we observed accuracies from 92.54 (BCFtools-SMALT) to 98.88 % (VarScan-Novoalign). Interestingly, the UnifiedGenotyper-SMALT combination resulted in the greatest numbers of true positive calls when either the high or low coverage datasets were aligned to the strain EGD-e chromosome. Furthermore, the VarScan-Novoalign combination resulted in the highest accuracy when the low coverage dataset was aligned to either the strain 08-5578 or EGD-e chromosomes.

We further analyzed the high coverage data by aligning the reads to both strain 08-5578 and EGD-e chromosome sequences before and after read quality filtering and trimming (Additional files 4, 5). When reads were aligned using the strain 08-5578 chromosome sequence, we observed no differences in the ability of SNP callers to identify nucleotide differences with and without trimming (Additional file 4A). We did observe substantial differences in the numbers of false positive calls before and after trimming. We witnessed between 11 and 210 false positive calls before trimming and from 11 to 73 such calls after read quality trimming and filtering (BCFtools-Novoalign and UnifiedGenotyper-BWA, respectively, in both cases; Additional file 4B). However, whether trimming resulted in a decrease or increase in the numbers of false positive sites called depended heavily upon the combinations of assembler and SNP caller. Using the UnifiedGenotyper-BWA combination to assemble and analyze reads after trimming resulted in a 65.24 % reduction in false positive calls, while using FreeBayes-MOSAIK to assemble reads after trimming caused a 54.55 % increase in the number of false positive calls (Additional file 6A). The highest accuracy (25.00 %) was achieved when using the BCFtools-MOSAIK combination without read quality trimming or filtering (Additional file 4C).

When the strain EGD-e chromosome sequence was used to align reads from the high coverage dataset we observed between 13,338 and 24,159 true positive sites identified prior to read quality filtering and trimming and from 13,304 and 24,164 after (FreeBayes-Novoalign and UnifiedGenotyper-SMALT, respectively, in each case; Additional file 5A). In addition, the numbers of false positive sites identified ranged from 167 and 2054 prior to trimming and from 168 to 2032 after (Additional file 5B). We also observed that the proportion of true positive calls to total calls ranged from 92.24 to 98.75 % (Additional file 5C). Again, whether read quality trimming and filtering provided any benefit depended heavily upon the assembler and SNP caller combinations. We noticed that read trimming had very little influence on the detection of true positives, no matter which combination of was used. However, when preprocessed reads were assembled and analyzed with FreeBayes-SMALT we observed a drop in false positive sites of approximately 20.68 % and when FreeBayes-MOSAIK was used we calculated an 11.25 % increase in the numbers of false positive calls (Additional file 6B).

In order to determine processing times for all combinations of reference-guided sequence assemblers and SNP callers assessed during this study, we assembled and reads obtained from a single run with an estimated 40-fold coverage using the strain EGD-e chromosome sequence as a reference (Additional file 7). We observed a wide range of processing times from approximately 152 s (BCF-SMALT) to 1440 s (VarScan-Novoalign) and an average processing time of 834 s for all combinations.

## Conclusions

Despite the fact that next-generation sequencing (NGS) technologies and open-source software have made comprehensive sequencing and single-nucleotide polymorphism (SNP) analysis of bacterial genomes accessible to individual laboratories, it is often unclear which reference-guided sequence assemblers and SNP callers should be used and what conditions will yield the most reliable results. NGS platforms typically generate millions of short sequence reads (for example, Illumina yields reads from 200 to 225 bp in length) that may contain inherent sequencing errors associated with specific sequencing technologies or the quality of DNA extractions and library preparations. The placement of these reads must then be accurately determined by assemblers that calculate the probability of its match with reference sequences that are usually megabases in length. Thus, genome sequence assembly is a formidable computational challenge that can be influenced by several factors, including: (1) the amount sequence coverage, (2) the algorithm used by the reference-guided sequence assemblers to place each read, (3) the distance of the reference sequence from the subject, and (4) whether read quality trimming and filtering has been performed. We have shown that each of the conditions listed here profoundly affected the performance of SNP callers, illustrating the complex relationship between genome sequence assembly and the accurate identification of nucleotide differences.

We found that, although true positive calls tend to increase with more sequencing coverage as one would expect, under some conditions false positive calls actually increased with higher coverage (Figs. 1, 2 and Additional files 1, 2: Tables S1, S2). Importantly, this phenomenon was observed when either the strain 08-5578 or the more distant EGD-e chromosome sequences were used as references. For example, assemblies generated with the Burrows-Wheeler aligner (BWA) and the strain 08-5578 reference sequence that were analyzed with VarScan generated such a trend, as did the use of BWA with the strain EGD-e reference when either the UnifiedGenotyper or VarScan were used. Interestingly, when we estimated the accuracy of SNP callers by calculating the proportions of calls made that correctly identified true positive sites, VarScan consistently outperformed other SNP callers when the low coverage dataset was analyzed (Figs. 1, 2, 3, Additional files 1, 2, 3: Tables S1, S2, S3). However, this was despite the fact that VarScan often reported the fewest numbers of true positive calls at low coverage and was due to reduced numbers of false positive calls. We observed also that assembling either low or high coverage data with SMALT using the strain EGD-e chromosome sequence as a reference and making SNP calls with the UnifiedGenotyper resulted in the greatest numbers of true positive calls (Fig. 3, Additional file 3). However, these conditions also resulted in the lowest (in the case of the high coverage dataset) or nearly the lowest (with the low coverage dataset) accuracy measurements, due to the high numbers of false positive calls. Finally, we have shown that whether read quality trimming and filtering provides benefit depends upon the combinations of assemblers and SNP callers used, in addition to one’s selection of reference sequence (Additional file 6).

In summary, we have revealed here an extraordinarily complex relationship between short read sequence assembly and SNP calling. The combinations of software tested here under a variety of conditions resulted in different numbers of true and false positive calls and different levels of accuracy (Additional files 4, 5: Figures S1, S2). This insight into the behaviors of SNP callers is useful for making informed decisions when designing experiments. It is important to note that we were unable to eliminate these tendencies by using either internal or external SNP filters; we observed that when false positive SNPs were filtered there was also a reduction (albeit non-linear) in the numbers of true positive calls as well. Therefore, researchers may need to consider whether detecting the greatest numbers of true positive sites, reducing the numbers of false positive calls, or achieving the highest levels of accuracy are in their best interest. And, it may be important to assess the abilities of different combinations of assemblers and SNP callers under various conditions in order to attain the most relevant results.

## Methods

### DNA extraction, library construction, and DNA sequencing

A *Listeria monocytogenes* strain HPB5622 isolate frozen in glycerol was streaked on pre-warmed Tryptose Agar plates and incubated at 37 °C over-night. A single colony was picked and used to inoculate 5 ml pre-warmed Brain Heart Infusion (BHI) broth and incubated over-night at 37 °C with shaking (200 rpm). Then, 200 µl of the culture was transferred to 50 ml pre-warmed BHI and incubated at 37 °C with shaking for 6 h to achieve the mid-logarithmic growth phase [35, 36]. Approximately 25 ml of culture was decanted into a 50 ml falcon tube and centrifuged at 3800 RCF for 5 min. The pellet was completely dissolved in 500 µl Tris-ethylenediaminetetraacetic acid by vortexing. We added 500 µl phenol–chloroform (1:1), 30 µl sodium acetate (3 M, pH 5.2), and 30 µl sodium dodecyl sulfate and mixed vigorously by shaking. The entire mixture was then pipetted into a 2 ml screw-cap tube filled with approximately 0.5 ml glass beads (0.1 mm). The tube was shaken in a Mini-Beadbeater machine (BioSpec products, Bartlesville, Oklahoma) for 45 s using the “Homogenizer” setting and placed on ice for 45 s. Shaking was repeated an additional four times. Approximately 300 µl of the mixture was then added to a Maxwell 16 Cell DNA Purification Kit cartridge and the sample was run using the standard DNA Blood/Cells protocol on a Maxwell 16 machine (Promega, Madison, Wisconsin) with elution in 300 µl nuclease-free water. RNA contamination was removed by adding 2 µl RNase A (Qiagen Sciences, Maryland) and incubating the sample for 10 min at 37 °C. A single phenol–chloroform-isoamyl alcohol (25:24:1) extraction followed by two ethanol precipitations was done. The sample was split into four subsamples. Subsamples were sequenced as previously described [37–39]. Briefly, each subsample was indexed with Nextera XT DNA Sample Preparation Kits (Illumina, San Diego, CA, USA) according to the standard protocol and sequenced (2 × 250 bp reads) on a MiSeq benchtop sequencer (Illumina) three separate times for a total of twelve sets of short-read sequences. These data have been deposited to the National Center for Biotechnology Information (NCBI) Sequence Read Archive (SRA) under accession numbers SRR1342176, SRR1342220, SRR1373524, SRR1373525, SRR1373534, SRR1373535, SRR1507228, and SRR1508282.

### Assembly of short-read sequence data

In order to ensure that only the highest quality data was used for assembly, reads were trimmed and filtered with PoPoolation set to a minimum length of 50 bp and a quality score threshold of 20. Global mapping of reads was then performed with each of four reference-guided short-read sequence assemblers: Burrows-Wheeler aligner v0.6.1-r104, MOSAIK v2.1 (code.google.com/p/mosaik-aligner/), Novoalign v3.00.03 (novocraft.com/main/index.php), and SMALT v0.7.4 (sanger.ac.uk/resources/software/smalt/). We used the Genome Analysis Toolkit [33] to perform local realignments around indels according to GATK best practices [40]. Single nucleotide polymorphisms were then identified with BCFtools (BCF) [30], FreeBayes [31], the UnifiedGenotyper (UGT; https://www.broadinstitute.org/gatk/gatkdocs/org_broadinstitute_gatk_tools_walkers_genotyper_UnifiedGenotyper.php), and VarScan [32, 33].

## Additional files

10.1186/s13104-015-1689-4 Calls made by SNP callers from alignments of reads to a nearly identical reference.
10.1186/s13104-015-1689-4 Calls made by SNP callers from alignments of reads to a non-identical reference.
10.1186/s13104-015-1689-4 The numbers of true and false positive SNPs detected with 16 combinations of sequence assemblers and SNP callers.
10.1186/s13104-015-1689-4 Calls made with 16 combinations of assemblers and SNP callers using a nearly identical reference.
10.1186/s13104-015-1689-4 Calls made with 16 combinations of assemblers and SNP callers using a non-identical reference.
10.1186/s13104-015-1689-4 Changes in the numbers of calls made after read quality trimming and filtering.
10.1186/s13104-015-1689-4 Processing times for assembly and SNP calling of a short-read dataset of approximately 40-fold coverage with 16 combinations of assemblers and SNP callers.

## Abbreviations

SNP: single-nucleotide polymorphism
LRS: Listeriosis reference service for Canada
BWA: burrows-wheeler aligner
NCBI: National Center for Biotechnology Information
BCF: BCFtools
UGT: UnifiedGenotyper
