# Supplementary material for: Choice of reference-guided sequence assembler and SNP caller for analysis of Listeria monocytogenes short-read sequence data greatly influences rates of error
Source: BMC Res Notes. 2015 Dec 8;8:748. doi: 10.1186/s13104-015-1689-4 (PMC4672502; doi:10.1186/s13104-015-1689-4)
Supplement: Supplementary file 2 — 10.1186/s13104-015-1689-4 Calls made by SNP callers from alignments of reads to a non-identical reference. [file 13104_2015_1689_MOESM2_ESM.pdf]

Additional File 2: Calls made by SNP callers from alignments of reads to a non-identical reference. The performance of four SNP callers was assessed by aligning 8 sets of reads to a reference chromosome that differs by 24,890 nucleotides and counting the numbers of false positive and true positive SNPs. The ranges in the numbers of events observed are shown with averages in parentheses. The values for all 8 datasets are provided as well as those at least 50-fold coverage. The best values for each category are bolded.

	True Positive Sites		False Positive Sites		(True Positive Sites/ Total Sites)*100	
	Total	≥ 50	Total	≥ 50	Total	≥ 50
BCFtools	16496-21363 (19985.38)	21359-21363 (21361.00)	424-799 (575.63)	424-477 (450.50)	95.90-98.05 (97.14)	97.82-98.05 (97.93)
FreeBayes	11651-13536 (12960.38)	13482-13536 (13509.00)	190-680 (301.13)	**206-213 (209.50)**	97.06-98.60 (97.69)	**98.44-98.50 (98.47)**
UGT	**17450-22156 (20692.13)**	**22092-22156 (22124.00)**	526-1052 (751.13)	961-1052 (1006.50)	95.47-97.42 (96.54)	95.47-95.83 (95.65)
VarScan	4340-19545 (14183.25)	19052-19545 (19298.50)	**53-489 (278.50)**	412-489 (450.50)	**97.56-98.83 (98.22)**	97.56-97.88 (97.72)
