# Supplementary material for: Choice of reference-guided sequence assembler and SNP caller for analysis of Listeria monocytogenes short-read sequence data greatly influences rates of error
Source: BMC Res Notes. 2015 Dec 8;8:748. doi: 10.1186/s13104-015-1689-4 (PMC4672502; doi:10.1186/s13104-015-1689-4)
Supplement: Supplementary file 3 — 10.1186/s13104-015-1689-4 The numbers of true and false positive SNPs detected with 16 combinations of sequence assemblers and SNP callers. [file 13104_2015_1689_MOESM3_ESM.pdf]

Additional File 3: True and false positive SNPs detected with 16 combinations of sequence assemblers and SNP callers. The best of eight Illumina sequencing runs was assembled with four assemblers and SNPs were identified in each assembly with four SNP callers.

	79-Fold / 08-5578											
	BCFtools			FreeBayes			UGT			VarScan		
	True	False	Percent	True	False	Percent	True	False	Percent	True	False	Percent
BWA	**3**	13	18.75	**3**	42	6.67	**3**	73	3.95	**3**	31	8.82
MOSAIK	**3**	12	**20.00**	**3**	68	4.23	**3**	39	7.14	**3**	53	5.36
Novoalign	2	**11**	15.38	2	44	4.35	2	31	6.06	1	23	4.17
SMALT	**3**	16	15.79	**3**	67	4.29	**3**	52	5.45	**3**	41	6.82

Additional File 3: True and false positive SNPs detected with 16 combinations of sequence assemblers and SNP callers – continued. The best of eight Illumina sequencing runs was assembled with four assemblers and SNPs were identified in each assembly with four SNP callers.

	79-Fold / EGD-e											
	BCFtools			FreeBayes			UGT			VarScan		
	True	False	Percent	True	False	Percent	True	False	Percent	True	False	Percent
BWA	21359	424	98.05	13536	206	98.50	22156	1052	95.47	19545	489	97.56
MOSAIK	22378	405	98.22	13937	267	98.12	22959	937	96.08	22113	585	97.42
Novoalign	19542	311	98.43	13304	**168**	**98.75**	19799	370	98.17	16892	239	98.60
SMALT	23933	1582	93.80	14057	441	96.96	**24164**	2032	92.24	23718	1364	94.56

Additional File 3: True and false positive SNPs detected with 16 combinations of sequence assemblers and SNP callers – continued. The best of eight Illumina sequencing runs was assembled with four assemblers and SNPs were identified in each assembly with four SNP callers.

	8-Fold / 08-5578											
	BCFtools			FreeBayes			UGT			VarScan		
	True	False	Percent	True	False	Percent	True	False	Percent	True	False	Percent
BWA	1	209	0.47	**3**	818	0.37	2	320	0.62	1	5	16.67
MOSAIK	1	93	1.06	**3**	885	0.34	1	226	0.44	1	11	8.33
Novoalign	2	148	1.33	2	640	0.31	2	293	0.68	1	**3**	**25.00**
SMALT	1	281	0.35	2	900	0.22	1	311	0.32	1	12	4.17

Additional File 3: True and false positive SNPs detected with 16 combinations of sequence assemblers and SNP callers – continued. The best of eight Illumina sequencing runs was assembled with four assemblers and SNPs were identified in each assembly with four SNP callers.

	8-Fold / EGD-e											
	BCFtools			FreeBayes			UGT			VarScan		
	True	False	Percent	True	False	Percent	True	False	Percent	True	False	Percent
BWA	16496	736	95.73	11651	680	94.49	17450	556	96.91	4340	**53**	98.79
MOSAIK	20719	387	98.17	13534	880	93.89	21901	592	97.37	9553	192	98.03
Novoalign	13239	512	96.28	10860	538	95.28	14692	370	97.54	4661	**53**	**98.88**
SMALT	22569	1820	92.54	13864	1084	92.75	**23504**	1611	93.59	11186	473	95.94
