# Supplementary material for: Choice of reference-guided sequence assembler and SNP caller for analysis of Listeria monocytogenes short-read sequence data greatly influences rates of error
Source: BMC Res Notes. 2015 Dec 8;8:748. doi: 10.1186/s13104-015-1689-4 (PMC4672502; doi:10.1186/s13104-015-1689-4)
Supplement: Supplementary file 5 — 10.1186/s13104-015-1689-4 Calls made with 16 combinations of assemblers and SNP callers using a non-identical reference. [file 13104_2015_1689_MOESM5_ESM.pdf]

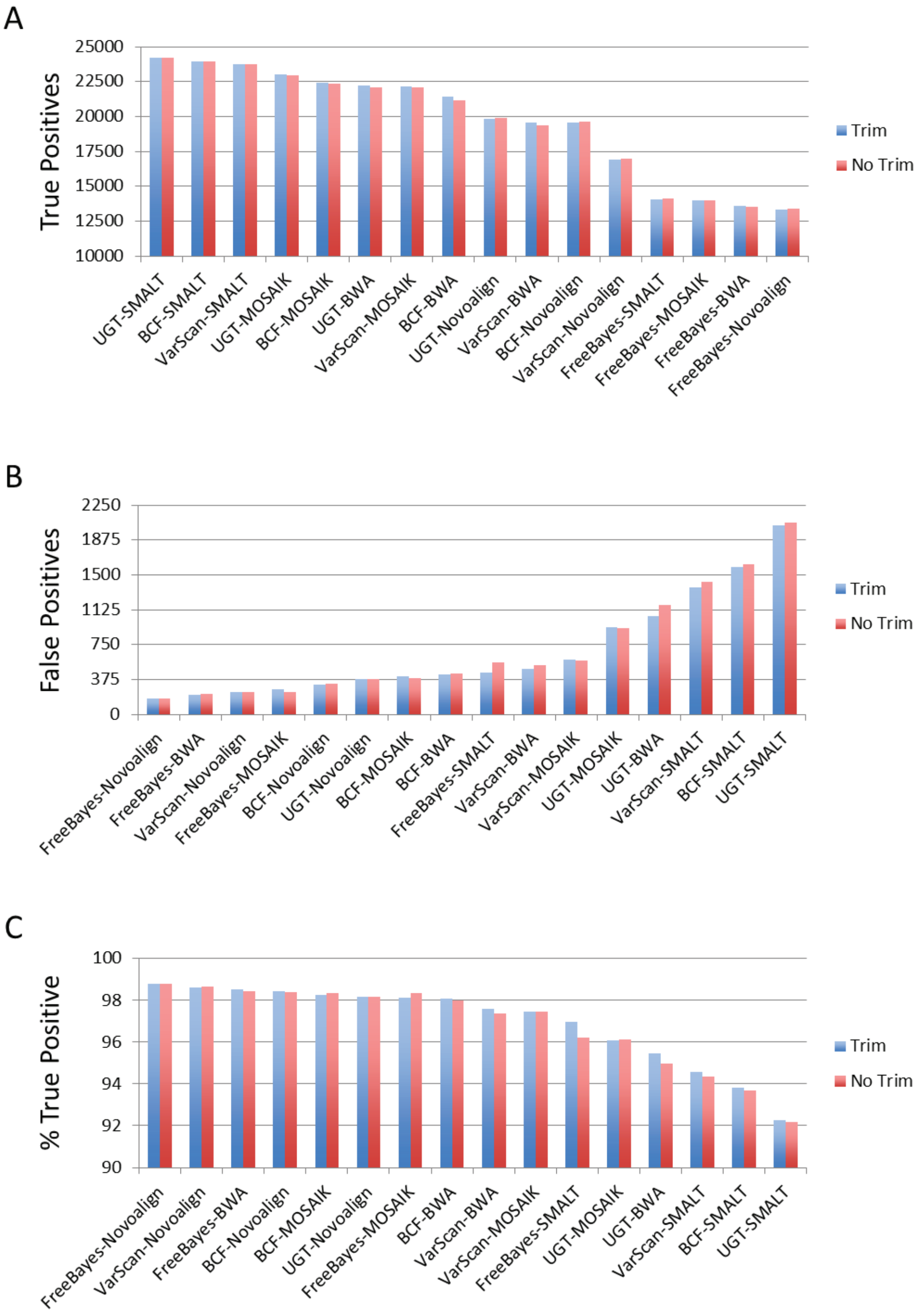

Additional File 5: Calls made with 16 combinations of assemblers and SNP callers using a non-identical reference. Genomic DNA from the Listeriosis Reference Service for Canada's (LRS) *Listeria monocytogenes* strain HPB5622 culture was indexed and sequenced to 79-fold coverage. The resulting reads were aligned with the Burrows-Wheeler Aligner (BWA), MOSAIK, Novoalign, and SMALT using an *L. monocytogenes* strain EGD-e chromosome sequence obtained from the National Center for Biotechnology Information (NCBI) archive as a reference. The EGD-e chromosome sequence differs from HPB5622 sequence at 24,890 nucleotide positions. Four SNP-callers (BCFtools [BCF], FreeBayes, UnifiedGenotyper [UGT], and VarScan) were used to identify nucleotide differences. The numbers of true positive calls (A), false positive calls (B), and the proportions of calls made that correctly identified true positive sites (C) relative to the calculated coverages of assemblies are shown. Values calculated before (No Trim) and after (Trim) read quality trimming and filtering are shown. Combinations are ranked by the "Trim" counts.
