# Supplementary material for: Choice of reference-guided sequence assembler and SNP caller for analysis of Listeria monocytogenes short-read sequence data greatly influences rates of error
Source: BMC Res Notes. 2015 Dec 8;8:748. doi: 10.1186/s13104-015-1689-4 (PMC4672502; doi:10.1186/s13104-015-1689-4)
Supplement: Supplementary file 6 — 10.1186/s13104-015-1689-4 Changes in the numbers of calls made after read quality trimming and filtering. [file 13104_2015_1689_MOESM6_ESM.pdf]

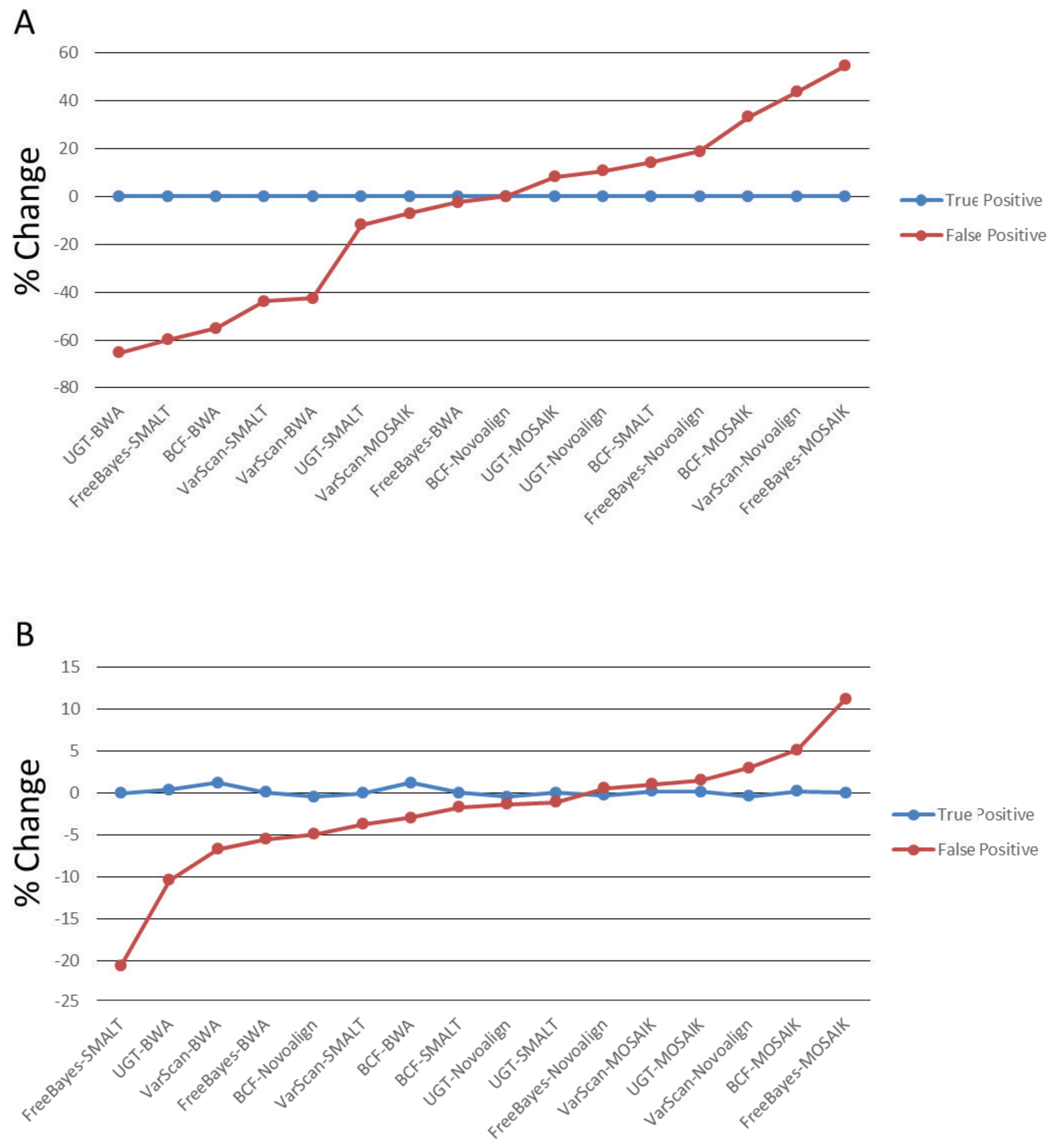

Additional File 6: Changes in the numbers of calls made after read quality trimming and filtering. Genomic DNA from the Listeriosis Reference Service for Canada's (LRS) *Listeria monocytogenes* strain HPB5622 culture was indexed and sequenced, yielding a high (~79-fold) and a low (~8-fold) coverage datasets. The resulting reads were aligned with the Burrows-Wheeler Aligner (BWA), MOSAIK, Novoalign, and SMALT using both *L. monocytogenes* strain 08-5578 (A) and EGD-e (B) chromosome sequences obtained from the National Center for Biotechnology Information (NCBI) archive as references. The NCBI strain 08-5578 chromosome sequence differs from HPB5622 at three nucleotide positions, while the EGD-e chromosome sequence differs at 24,890 nucleotide positions. Four SNP-callers (BCFtools [BCF], FreeBayes, UnifiedGenotyper [UGT], and VarScan) were used to identify nucleotide differences.
