# Supplementary material for: Choice of reference-guided sequence assembler and SNP caller for analysis of Listeria monocytogenes short-read sequence data greatly influences rates of error
Source: BMC Res Notes. 2015 Dec 8;8:748. doi: 10.1186/s13104-015-1689-4 (PMC4672502; doi:10.1186/s13104-015-1689-4)
Supplement: Supplementary file 7 — 10.1186/s13104-015-1689-4 Processing times for assembly and SNP calling of a short-read dataset of approximately 40-fold coverage with 16 combinations of assemblers and SNP callers. [file 13104_2015_1689_MOESM7_ESM.pdf]

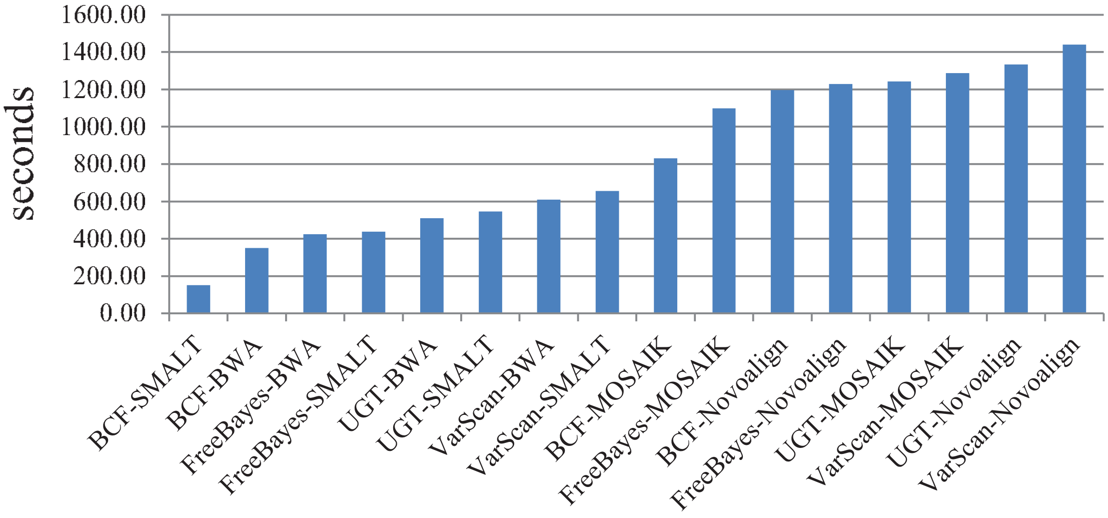

Additional File 7: Processing times for 16 combinations of assemblers and SNP callers. Genomic DNA from the Listeriosis Reference Service for Canada's (LRS) *Listeria monocytogenes* strain HPB5622 culture was indexed and sequenced. Reads from one run estimated at 40-fold coverage were aligned with the Burrows-Wheeler Aligner (BWA), MOSAIK, Novoalign, and SMALT using an *L. monocytogenes* strain EGD-e chromosome sequence obtained from the National Center for Biotechnology Information (NCBI) archive as a reference. SNPs were called with BCFtools [BCF], Freebayes, UnifiedGenotyper [UGT], and VarScan. Analyses were performed with an AMD Phenom II X6 1090T processor and 16 GB of DDR3 RAM.
